# Modelling the Effects of Ageing Time of Starch on the Enzymatic Activity of Three Amylolytic Enzymes

**DOI:** 10.1100/2012/402439

**Published:** 2012-02-09

**Authors:** Nelson P. Guerra, Lorenzo Pastrana Castro

**Affiliations:** Department of Analytical and Food Chemistry, Food Science and Technology Faculty, University of Vigo, Ourense Campus, 32004 Ourense, Spain

## Abstract

The effect of increasing ageing time (*t*) of starch on the activity of three amylolytic enzymes (Termamyl, San Super, and BAN) was investigated. Although all the enzymatic reactions follow michaelian kinetics, *v*_max_ decreased significantly (*P* < 0.05) and *K*_*M*_ increased (although not always significantly) with the increase in *t*. The conformational changes produced in the starch chains as a consequence of the ageing seemed to affect negatively the diffusivity of the starch to the active site of the enzymes and the release of the reaction products to the medium. A similar effect was observed when the enzymatic reactions were carried out with unaged starches supplemented with different concentrations of gelatine [*G*]. The inhibition in the amylolytic activities was best mathematically described by using three modified forms of the Michaelis-Menten model, which included a term to consider, respectively, the linear, exponential, and hyperbolic inhibitory effects of *t* and [*G*].

## 1. Introduction

Starch is composed of two high-molecular-weight components: amylose, a linear polymer consisting of D-glucose units linked together through *α*(1→4) glycosidic bonds, and amylopectin, a highly branched polymer, which consists of linearly *α*(1→4) linked D-glucose units and *α*(1→6) linked D-glucose units which provide branching points [1, 2]. This heterogeneous polysaccharide is hydrolyzed by amylolytic enzymes [1], including endoamylases, exoamylases, and debranching enzymes [2]. 

The enzymes belonging to the first group, the *α*-amylases (EC 3.2.1.1, *α*-D-glucan glucanohydrolase), cleaves the *α*(1→4) glycosidic bonds of the substrate (amylose or amylopectin chain) at internal positions (endo) to yield products (oligosaccharides with varying length an branched oligosaccharides called limit dextrins) with an *α*-configuration [2, 3].

Two important amylolytic enzymes belong to the group of exoamylases: *β*-amylase (EC 3.2.1.2, *α*-D-glucan maltohydrolase) and glucoamylase (EC 3.2.1.3, *α*-D-glucan glucohydrolase). The first enzyme hydrolyzes maltosyl units from the nonreducing end of amylose or amylopectin to yield maltose in the *β*-configuration and *β*-limit dextrin. The glucoamylase catalyses the hydrolysis of both *α*(1→4) and *α*(1→6) glycosidic bonds at the branching point to release *β*-D-glucose units successively from the nonreducing external glucose residues of the polymer substrate [2–5].

Starch and the hydrolysis products of it (Maltodextrin and maltose) have been widely used as food ingredients in the food industry [6].

Activity of Michaelian enzymes is satisfactorily described by the classical Michaelis-Menten model:

(1) $$v=\frac{v_{\max}\cdot \left[S\right]}{K_{M}+\left[S\right]},$$

where *v* and *v*_max⁡_ are, respectively, the rate and the maximum rate of substrate conversion, [*S*] is the initial starch concentration, and *K*_*M*_ is the Michaelis constant.

Velocity of enzyme catalysed reactions can be reduced in presence of nonspecific irreversible inhibitors (e.g., urea or high temperatures) or molecules that produce reversible inhibitions that fall into the following categories: competitive, pure or mixed noncompetitive and uncompetitive. The nonspecific inhibition is related with any physical or chemical changes which cause an irreversible denaturation of the protein portion of the enzyme and consequently enzyme stops to work. In the reversible competitive inhibition, the inhibitor competes with the substrate for binding to the active site of the enzyme. As a consequence, the rate at low [*S*] decreases and the apparent *K*_*M*_ increases. This inhibition can be overcome with the increase in substrate concentration. The pure noncompetitive inhibitor binds to the enzyme, but at a site away from the active site, producing a reduction in the value of *v*_max⁡_ but the *K*_*M*_ value is unchanged. In this case, the inhibitor has an identical affinity for the enzyme and the enzyme-substrate complex. Its action changes the shape of the enzyme and thus the active site, limiting the interaction between the enzyme and the substrate. In contrast, a mixed noncompetitive inhibitor has a different affinity for both the free enzyme and the enzyme-substrate complex. The uncompetitive inhibition takes places when the inhibitor binds to the enzyme-substrate complex but not to the enzyme. As a consequence, the values of *v*_max⁡_ and *K*_*M*_ decreased [7, 8].

However, the rates of the enzymatic reactions could also be affected when a physical factor produces conformational changes in the substrates, such as those produced by the physical ageing of starch [9].

Physical ageing is a phenomenon that occurs when a glassy polymer, which initially is not in a thermal equilibrium, relaxes toward an equilibrium under a kinetic control [9, 10]. Some changes in the physical and mechanical properties of the glassy polymer are produced during this process, including the increase in the glass transition temperature, relaxation enthalpy, and storage modulus [11] and a progressive stiffening and embrittlement of the polymer [12]. In case of starch, these structural rearrangements in amorphous matrix produces a reduction in the mobility of the amylose and amylopectin chains and an increase in the density of the matrix and, consequently the Michaelian parameters of amylolytic enzymes could be affected [11]. However, to our knowledge, there are no reports dealing with the effect of ageing time of starch on the activity of amylolytic enzymes.

Therefore, the main goal of the present study was to determine the effects of ageing time (0, 24, 48, and 100 h) of starch on the Michaelin behaviour of three amylolytic enzymes (Termamyl, San Super, and BAN). From the experimental data obtained, the Michaelian parameters *v*_max⁡_ and *K*_*M*_ in each enzymatic reaction were calculated to classify the inhibition type produced by the ageing time. The following experiment was carried out to determine if the changes in the Michaelian parameters were produced by a limitation in diffusivity of the starch from the medium to the active site of the enzymes and the release of the reaction products from the active site to the medium. Thus, the susceptibility of unaged starch to enzymatic degradation by the three amylolytic enzymes was study in presence of different gelatine concentrations (0, 25, 50, and 75 g/L). The effects of ageing time and gelatine concentrations on the three amylolytic enzymes were then modelled by using three modified forms of the classical Michaelis Menten model developed in this study.

## 2. Materials and Methods

### 2.1. Enzymes

The three preparations of amylase enzymes used in this work were Termamyl 120L (L), an *α*-amilasa from *Bacillus licheniformis*, San Super 240L, a glucoamylase from *Aspergillus niger*, and BAN 240L, an *α*-amilasa from *Bacillus amyloliquefaciens*. The enzyme preparations were supplied by Novo Nordisk A/S Industries, (Bagsvaerd, Denmark).

### 2.2. Starch Preparation for Ageing Experiments

Starch was purchased from Panreac Química S.A. (Barcelona, Spain). This substrate was firstly suspended in a convenient volume of distilled water with vigorous stirring to obtain a homogeneous and transparent suspension. Subsequently, the starch suspension was poured into a volume of boiling distilled water, and after vigorous stirring, the clear solution was cooled and made up to the final volume with distilled water. To perform the ageing, the starch solutions were allowed to stand for 24, 48, or 100 h at room temperature. These different aged starches were then used to study the effect of ageing time on the enzymatic activity of the three amylase enzymes.

### 2.3. Experiments in Unaged Starch Mixed with Different Initial Concentrations of Gelatine

Gelatine from porcine skin was purchased from Sigma-Aldrich Co. (St. Louis, USA). For the experiments with this protein, appropriate amounts of unaged starch suspensions of various concentrations were mixed with increasing gelatine concentrations (25, 50, and 75 g/L). A control experiment was carried out by using unaged starch without gelatine prepared conveniently to obtain the same starch concentrations as the substrates mixed with gelatine. These samples were then used as substrates in the amylolytic enzyme assays.

### 2.4. Enzyme Assays

Total amylase activity was determined according to Murado et al. [13], mixing 80 *μ*L of each amylase enzyme preparation (suitably diluted) with 400 *μ*L of 0.15 M citrate-phosphate buffer; pH 5.0 (1 volume), and 4% soluble starch (1.5 volumes) previously maintained at 40°C/15 min. The reaction mixture was incubated at 40°C for 10 min. The reaction was stopped by addition of 480 *μ*L of dinitrosalicylic acid, and the released glucose was determined by 3,5-dinitrosalicylic acid reaction [14]. One unit of amylase activity was defined as the amount of enzyme that releases 1 mg/mL of reducing sugars (glucose equivalents) under the assay conditions.

### 2.5. Statistical Analyses

Individual experiments were performed in triplicate and the analytical determinations (reducing sugars) were performed in duplicate, with the experimental results being presented as mean ± standard deviations. Data sets were statistically analyzed by using the software package SPSS Statistics 17.0 for Windows (Release 17.0.1; SPSS Inc., Chicago, IL, 2008). A *t*-test was conducted to determine whether significant differences at the 95% level (*P* < 0.05) existed between the amylase activities obtained in unaged starch and in starches aged for different times (24, 48 and 100 h) and in unaged starches mixed with gelatine concentrations of 0, 25, 50, and 75 g/L. The same statistical test was used to compare the values of the parameters *v*_max⁡_ and *K*_*M*_ obtained after modelling the enzymatic activity of the three amylases with the corresponding models.

### 2.6. Model Parameters Determination and Model Evaluation

The model parameters were obtained by using the nonlinear curve-fitting software of SigmaPlot (version 9.0, Systat Software, Inc., 2004), which minimized the deviations between model predictions and experimental data according to the sum of squares of errors (SSE) of the model fit:

(2) $$\begin{array}{cc} \text{SSE} & =\overset{n}{\underset{i=1}{\sum }}\overset{m}{\underset{j=1}{\sum }}\Delta_{i,j}^{2}=\overset{n}{\underset{i=1}{\sum }}\overset{m}{\underset{j=1}{\sum }}{\left(v_{\exp}-v_{\text{pred}}\right)}^{2}\\ & ={\overset{n}{\underset{i=1}{\sum }}\overset{m}{\underset{j=1}{\sum }}\left(v_{\exp}-\frac{v_{\max}\cdot \left[S\right]}{K_{M}+\left[S\right]}\right)}^{2}, \end{array}$$

where Δ_*i*,*j*_ represents the difference between the velocity predicted by the model (*v*_pred_) and the experimental velocity value (*v*_exp⁡_), *n* and *m* represent the number of experimental data points, and the number of variables, respectively. The other variables were defined above in model (1).

The coefficients of the models with *P* values lower than 0.05 were considered statistically significant. Parameters were removed from the models when their asymptotic interval of confidence included zero.

The criteria used to evaluate the goodness of fit of each model were the determination coefficient (*R* ^2^) and the mean relative percentage deviation modulus (RPDM) [15]:

(3) $$\text{RPDM}=\frac{100}{N}\overset{N}{\underset{i=1}{\sum }}\frac{\left|X_{i}-X_{pi}\right|}{X_{i}},$$

where *X*_*i*_ is the experimental value, *X*_*pi*_ is the calculated value, and *N* is the number of experimental data. A value of RPDM below 10% is indicative of a good fit for practical purposes [15–17].

## 3. Results and Discussion

### 3.1. Effect of the Physical Ageing of Starch on the Michaelian Parameters of the Amylases Enzymes Termamyl, San Super, and BAN

The first experiment was conducted to determine the effect of the ageing time of starch (0, 24, 48, and 100 h) in the kinetic behaviour of the amylolytic enzymes Termamyl, San Super, and BAN. Subsequently, the experimental enzymatic activities of the three enzymes were modelled with the Michaelis-Menten model (1).

**Figure 1:**
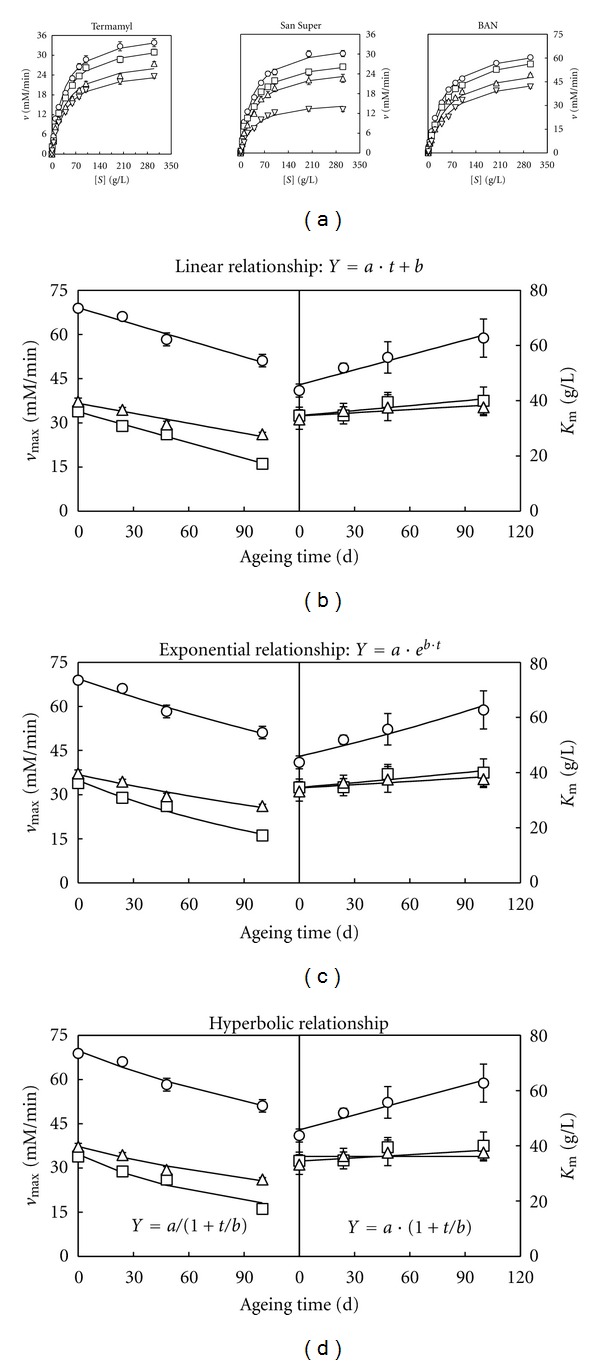
(a) Changes in the experimental amylolytic activity (*v*) data (means ± standard deviations) with the concentration of unaged starch (O) and starches [*S*] aged for 24 h (□), 48 h (∆), and 100 h (∇). The curves drawn through the experimental velocity data in (a) were obtained according to the Michaelis-Menten model (1). The curves drawn through the *v*_max⁡_ and *K*_*M*_ data (as means ± standard errors) for the enzymes BAN (O), San Super (□), and Termamyl (∆) were obtained according to a linear (b), exponential (c), and hyperbolic (d) equation. *Y* represents the predicted values for *v*_max⁡_ and *K*_*M*_, *a* and *b* are constants, and *t* is the ageing time.

The results obtained (Figure 1(a), Table 1) showed that all the enzymes followed a Michaelian behaviour with independence of the ageing time, because significant values (*P* < 0.05) for the *v*_max⁡_ and *K*_*M*_ were obtained in each case, with *R* ^2^ values higher than 0.98 and RPDM values lower than 10% (Table 1). Thus, excellent agreement was found between model predictions and experimental results for the amylolytic enzymes Termamyl, San Super, and BAN.

However, from the detailed observation of the results obtained it can be noted that, for the these enzymes, the values obtained for the parameters *v*_max⁡_ decreased significantly (*P* < 0.05), and *K*_*M*_ increased (although not always significantly) with the increase in the ageing time (*t*) of starch (Table 1). Then, the values of *v*_max⁡_ and *K*_*M*_ become apparent for *t* ≠ 0.

The observed reduction in the amylolytic activities of the three enzymes was probably due to the conformational changes produced in the starch chains during ageing, which were produced by a decrease in the free volume and mobility of the constituent amylase and amylopectin chains and an increase in the rigidity of the matrix [11]. Then, the new degree of organization of the amylose and amylopectin chains in the rigid matrix after the starch was aged, probably affected negatively the interactions between substrate and active site of the amylolytic enzymes and the stabilization of the productive enzyme/substrate complex [18].

**Figure 2:**
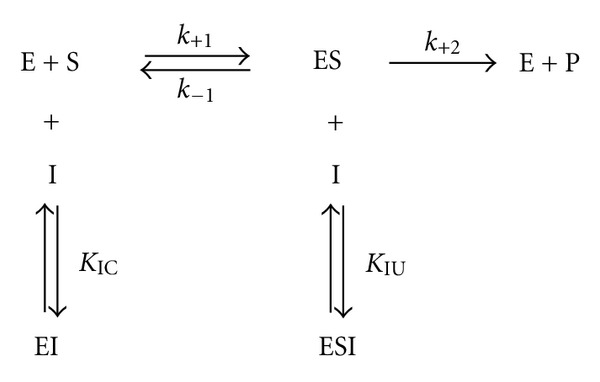
Scheme depicting the mixed competitive inhibition mechanism for the interaction of an inhibitor molecule (*I*) and an enzyme (*E*) in presence of substrate (*S*). *k*_+1_, *k*_−1_, and *k*_+2_ are rate constants. *K*_IC_ and *K*_IU_ are, respectively, the competitive and the uncompetitive constants for the EI and ESI complexes.

On the other hand, the results showed in Table 1 suggest that the ageing time of starch produced an inhibitory effect similar to that produced by a mixed competitive inhibitor, because *v*_max⁡_ decreased, *K*_*M*_ increased and the ratio *v*_max⁡_/*K*_*M*_ decreases as *t* increased. However, the inhibition mechanism of a mixed competitive inhibitor (Figure 2) [19, 20] is quite different to that produced by a physical factor such as the ageing time or an external mechanical force [21].

On the one hand, the mixed inhibition produced by an inhibitor molecule refers to a combination of the competitive and uncompetitive inhibition, which are two different types of reversible enzyme inhibition. In this case, the inhibitor can bind to either the free enzyme (*E*) or the enzyme-substrate complex (ES) by a site different from the active site where the substrate binds. However, the inhibitor affinity for *E* and ES is different (*K*_IC_ ≠ *K*_IU_). Since a mixed-type inhibitor interferes with substrate binding and hamper the catalysis in the ES complex, a decrease in the apparent affinity of the enzyme for the substrate (*K*_*M*_^app^ > *K*_*M*_) and in the apparent maximum enzyme reaction rate (*v*_max⁡_^app^ < *v*_max⁡_) is produced [22, 23].

On the other hand, the inhibition produced by the ageing time on the activity of the amylolytic enzymes could be related to its action on the conformation of the substrate. In consequence, the inhibition produced by this physical factor cannot be described by using the mechanistic model (4), which describes the effects of a mixed competitive inhibitor:

(4) $$v=\frac{v_{\max}\cdot \left[S\right]}{K_{M}\cdot \left(1+\left[I\right]/K_{\text{IC}}\right)+\left[S\right]\cdot \left(1+\left[I\right]/K_{\text{IU}}\right)},$$

where *K*_IC_ and *K*_IU_ are the competitive and the uncompetitive constants for the EI and ESI complexes and [*I*] is the inhibitor concentration. Other terms are as previously described.

Therefore, it seems more adequate to develop a modified form of the Michaelis Menten model (1) to describe the effect of the ageing time of starch on the activity of the enzymes Termamyl, San Super, and BAN.

However, before modifying the Michaelis-Menten model, it is necessary to classify at what type of inhibition the ageing time of starch belongs. The specific type of inhibition can be determined by plotting the calculated *v*_max⁡_ and *K*_*M*_ values against the ageing time to see how the increase in *t* affects the two Michaelian parameters. The results obtained (Figures 1(b), 1(c) and 1(d)) suggest that the relationship between the Michaelian parameters *v*_max⁡_ and *K*_*M*_ with the ageing time could be linear (Figure 1(b)), exponential (Figure 1(c)), or hyperbolic (Figure 1(d)). Thus, considering these three situations, the resulting modified model (1) becomes

(5) $$v=\frac{v_{\max}\cdot \left[S\right]}{K_{M}+\left[S\right]}\cdot \left(1-K_{t}\cdot \left[t\right]\right),$$

(6) $$\begin{array}{c} v=\frac{v_{\max}\cdot \left[S\right]}{K_{M}+\left[S\right]}\cdot e^{\left(-K_{t}\cdot [t]\right)} \end{array},$$

(7) $$v=\frac{v_{\max}\cdot \left[S\right]}{\left(K_{M}+\left[S\right]\right)}\cdot \left(\frac{K_{t}}{K_{t}+t}\right),$$

where *K*_*t*_ is the inhibition constant for the ageing time of starch (in days^−1^ for models (5) and (6) or in days for model (7)), and *t* is the ageing time (days) of starch. Other terms are as previously described.

**Figure 3:**
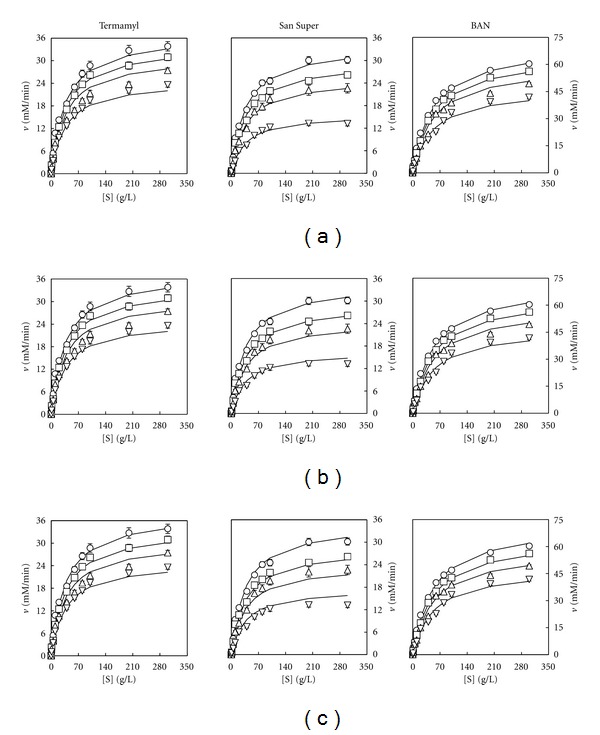
Changes in amylolytic activity (*v*) with the concentration of unaged starch (O) and starches [*S*] aged for 24 h (□), 48 h (∆), and 100 h (∇). The experimental points (as means ± standard deviations) are the amylolytic activity data showed in Figure 1(a) for each enzyme. The curves drawn through the experimental velocity data in (a), (b), and (c) were obtained according to the models (5), (6), and (7), respectively.

The trajectories described by the overall models (5), (6), and (7) (solid lines in Figures 3(a), 3(b), and 3(c) indicated an excellent agreement between model predictions and experimental results. In addition, the high *R* ^2^ values (>0.98) and the RPDM values lower than 10% (Table 2) have strengthened the usefulness of the proposed overall models (5), (6), and (7) for describing the enzymatic activity of the three amylolytic enzymes on starches aged for different times. 

On the other hand, it can be noted that for each enzyme, the three overall models provided similar values (*P* < 0.05) not only for the constants *v*_max⁡_ and *K*_*M*_ but also for the *R* ^2^ coefficient, RPDM, and SEE (Table 2).

In an attempt for determining what model describes more accurately the trend of the data, the calculated values of *v*_max⁡_ and *K*_*M*_ obtained with the models (5), (6), and (7) were compared with those obtained with model (1) for the enzymatic reactions in unaged starch (*t* = 0 h). The results (Table 3) showed that there were no significant differences (*P* < 0.05) between the Michaelian parameters, with the exception of the *K*_*M*_ value obtained with model (6) for the BAN enzyme. This observation corroborates the capability of the three modified models for describing adequately the enzymatic activity of the amylolytic enzymes and it makes difficult the selection of the most adequate model.

Then, the model (5), which has the simplest mathematical expression, was used to simulate the amylolytic activity (response variable) of the Termamyl, San Super, and BAN enzymes as a function of two independent variables: the ageing time and the substrate concentration.

**Figure 4:**
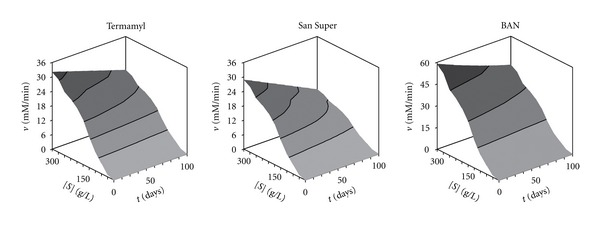
Response surfaces plot showing the effect of the ageing time (*t*) and the starch concentration [*S*] on the amylolytic activity (*v*) of the three enzymes.

The response surfaces generated with model (5), clearly showed that in all cases, the increase in substrate concentration led to a hyperbolic increase in the response, but the values of the latter variable decreased as the ageing time increased, mainly in case of San Super enzyme (Figure 4). This was due to the fact that the latter enzyme had the highest *K*_*t*_ value (Table 2).

### 3.2. Effect of the Addition of Gelatine to the Reaction Mixture on the Michaelian Parameters of Three Amylases Enzymes on Unaged Starch

In the former experiments, it was demonstrated that the increase in the ageing time of starch led to a modification in the Michaelian constants *v*_max⁡_ and *K*_*M*_. This was related with an increase in the starch crowding, that probably limited both the diffusivity of the starch from the medium to the active site of the enzymes and the release of the reaction products (reducing sugars) from the active site to the medium [11, 24].

In an attempt for corroborating this hypothesis, a new series of experiments was carried out in the same experimental conditions as those of the former assays. In these experiments, the substrates consisted of unaged starches mixed with different initial gelatine concentrations: 25, 50, and 75 g/L. Since the increase in gelatine concentration led to an increase in the medium viscosity [25], diffusivities of both the substrate and products are expected to decrease. In addition, the protein nature of gelatine makes it resistant to the amylases attack and consequently, this substance does not participate in the enzymatic reaction.

Taking into account that the addition of gelatine led to a decrease in starch concentration in the reaction mixture, a control experiment without gelatine was carried out for each enzyme, by using initial starch concentrations similar to those obtained in the experiments with gelatine.

**Figure 5:**
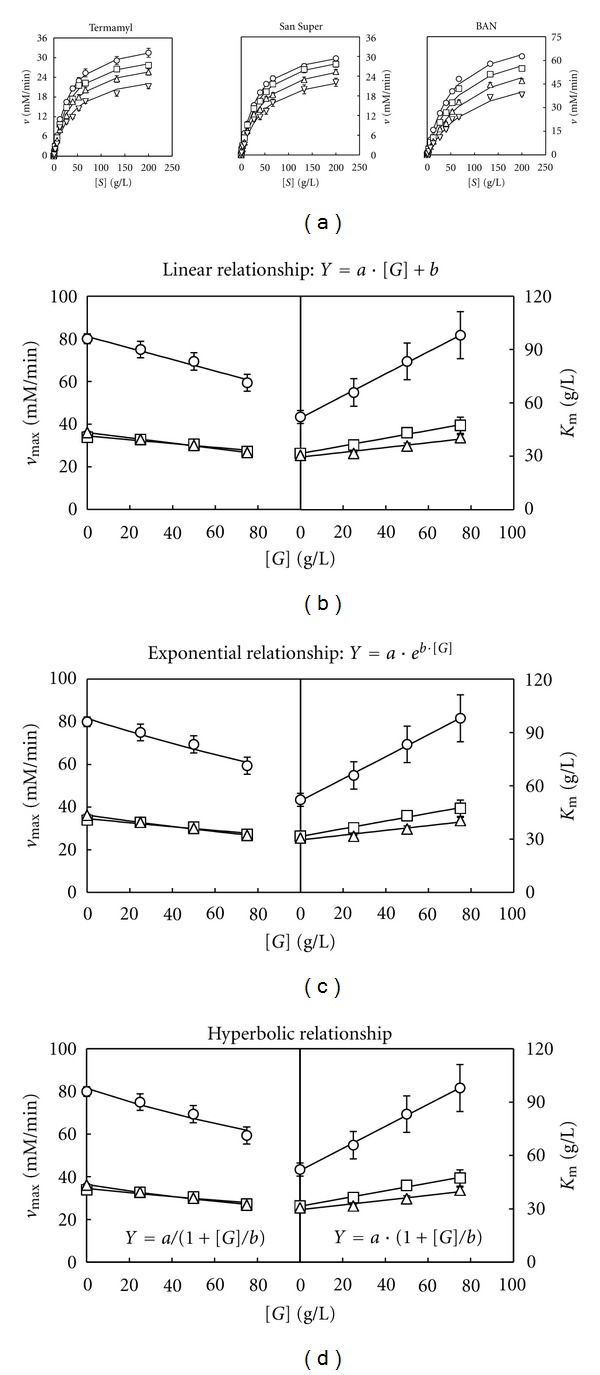
Changes in the experimental amylolytic activity (*v*) data (means ± standard deviations) with the concentration of starch (O) and starches mixed with initial gelatine concentrations [*G*] of 25 g/L (□), 50 g/L (∆), and 75 g/L (∇). The curves drawn through the experimental velocity data in (a) were obtained according to the Michaelis-Menten model (1). The curves drawn through the *v*_max⁡_ and *K*_*M*_ data (as means ± standard errors) for the enzymes BAN (O), San Super (□), and Termamyl (∆) were obtained according to a linear (b), exponential (c), and hyperbolic (d) equation. *Y* represents the predicted values for *v*_max⁡_ and *K*_*M*_, *a*, and *b* are constants.

The results obtained (upper part of Figure 5) showed that the three enzymes followed again a Michaelian behaviour as it was observed before in the reactions with aged starches (Figure 1). Thus, after using model (1) to describe the trend of the experimental amylolytic activity on different starch concentrations, significant values (*P* < 0.05) were obtained for the model parameters *v*_max⁡_ and *K*_*M*_ for each enzyme and each gelatine concentration (Table 4). 

As expected, the increase in gelatine concentration led to an increase in the *K*_*M*_ values and a decrease in *v*_max⁡_ values (Table 4). In fact, the results obtained showed a trend similar to that observed in the former experiment (Table 1), thus suggesting that the inhibitory effects of the increasing ageing time on the amylolytic activity could be related with a limitation to the mass transfer.

As it was assumed in the experiments with aged starches, the increasing gelatine concentrations were considered to produce a linear, exponential, or hyperbolic inhibition on the enzymatic activity of the Termamyl, San Super, and BAN enzymes (Figures 5(b), 5(c), and 5(d)).

Therefore, the three modified Michaelis Menten models (5), (6), and (7) were also used to describe the effects of increasing gelatine concentrations in amylolytic activity of the three enzymes. Before using these models, the inhibition constant *K*_*t*_ was changed by *K*_*G*_, which is now, the inhibition constant for the gelatine (in L/g for models (5) and (6) or in g/L for model (7)). In the same way, *t* was changed by [*G*], which is the gelatine concentration (g/L).

**Figure 6:**
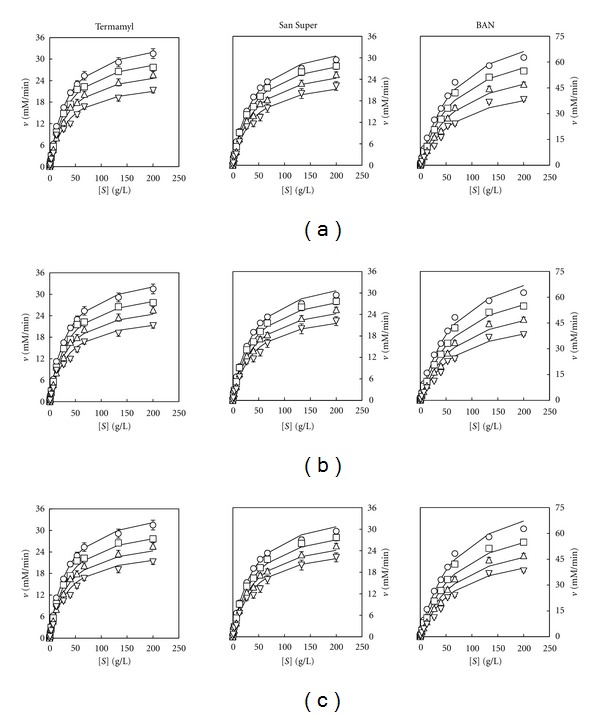
Changes in velocity (*v*) for each amylolytic enzyme with the concentration of starch (O) and starches [*S*] mixed with initial gelatine concentrations [*G*] of 25 g/L (□), 50 g/L (∆), and 75 g/L (∇). The experimental points (as means ± standard deviations) are the amylolytic activity data showed in Figure 5(a) for each enzyme. The curves drawn through the experimental velocity data in (a), (b), and (c) were obtained according to the models (5), (6), and (7), respectively.

The satisfactory agreement between the calculated curves and the experimental data points indicates that the three models were a suitable description of the behaviour of the enzymes (Figure 6). In addition, the high *R* ^2^ values (higher than 0.98), as well as the low values obtained for RPDM (lower than 10%) and SEE (Table 5), indicated that the proposed models can accommodate adequately the experimental data.

To evaluate the ability of the overall models (5), (6), and (7) for describing the trend of the experimental data, the values of the Michaelian parameters calculated with these models (Table 5) were compared with those obtained with the model (1) for the reactions without gelatine (Table 4). The results obtained (Table 6) showed a statistical significant agreement (*P* < 0.05) between the Michaelian parameters for the BAN enzyme. In fact, the values of *K*_*M*_ obtained with the three overall models were not statistically different to those obtained with model (1) for each enzyme. However, the value of *v*_max⁡_ obtained with model (1) was found to be significantly different to that calculated with the model (7) (in case of San Super) and to those calculated with the three overall models (in case of Termamyl). Taking into account the *P* values (Table 6) obtained for the latter enzyme (*P* = 0.045), it can be concluded that the *v*_max⁡_ value calculated with model (5) showed the lowest difference with the *v*_max⁡_ obtained with model (1). This result suggests that the amylolytic activity of Termamyl in presence of increasing concentrations of gelatine could be best mathematically described by model (5).

For this reason and taking into account the difficulty of finding significant differences between the three overall models (Table 5), we selected the model (5) to describe the effects of substrate and gelatine concentrations on the amylolytic activity of the three enzymes.

**Figure 7:**
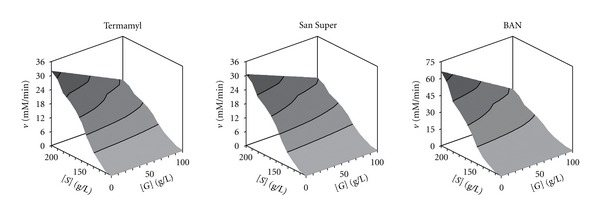
Response surfaces plot showing the effect of the concentrations of starch [*S*] and gelatine [*G*] on the amylolytic activity (*v*) of the three enzymes.

The response surfaces (Figure 7) generated with model (5) showed that the increase in gelatine concentration produced the highest inhibitory effect on the activity of the BAN enzyme, which had the highest value for the *K*_*G*_ constant (Table 5). From these response surfaces, it can be observed clearly that the maximum amylase activity for the three enzymes was located at high substrate concentrations [*S*] and low values of gelatine concentration [*G*].

## 4. Conclusion

The main contribution of this paper is the description of the kinetics of the amylolytic enzymes San Super, Termamyl, and BAN in starches aged for different times. The results showed that the three enzymes follow a Michelian behaviour with independence of the ageing time of the starch, which acted as an inhibitor of the activity enzymatic. The addition of increasing gelatine concentrations to the reaction mixture inhibited the activity of the amylolytic enzymes in a similar way as that produced by the ageing time. The effect of both inhibitors was successfully modelled by using three modified forms of the Michaelis-Menten model.

To our knowledge, it is the first time that the amylolytic activities of these three enzymes are modelled as a function of the increase in substrate concentration and the ageing time or gelatine concentration. The above-described mathematical models could be applied to operate and control the enzymatic hydrolysis of starch by the three amylolytic enzymes in an industrial bioreactor [26].

## Figures and Tables

**Table 1:** Statistically significant (*P* < 0.05) values (means ± standard errors) of the parameters in the Michaelis-Menten model (1) for the enzymatic reactions of the three amylolytic enzymes on starches aged for different times (0 h, 24 h, 48 h, and 100 h). The mean values within rows followed by the same letter are not significantly different (*P* < 0.05) from each other.

	Termamyl			
Parameters	*t* = 0 h	*t* = 24 h	*t* = 48 h	*t* = 100 h

*v*_ max_	37.2 ± 1.22a	34.4 ± 0.83b	29.4 ± 1.11c	26.1 ± 0.67d
*K*_*M*_	33.2 ± 3.55a	36.3 ± 2.76b	37.5 ± 4.67b,c	37.6 ± 3.05c,d
*R* ^2^	0.9916	0.9958	0.9886	0.9953
RPDM	9.71	8.09	9.59	7.78
*v*_max⁡_/*K*_*M*_	1.12	0.95	0.78	0.69

	San Super			

Parameters	*t* = 0 h	*t* = 24 h	*t* = 48 h	*t* = 100 h

*v*_max⁡_	33.9 ± 0.89a	28.9 ± 0.78b	26.1 ± 0.72c	16.1 ± 0.61d
*K*_*M*_	34.6 ± 3.06a	34.7 ± 3.10a,b	39.5 ± 3.49c	40.0 ± 4.96c,d
*R* ^2^	0.9945	0.9944	0.9946	0.9893
RPDM	5.50	6.53	5.76	6.30
*v*_max⁡_/*K*_*M*_	0.98	0.83	0.66	0.40

	BAN			

Parameters	*t* = 0 h	*t* = 24 h	*t* = 48 h	*t* = 100 h

*v*_max⁡_	68.9 ± 1.22a	66.1 ± 0.72b	58.3 ± 2.17c	51.1 ± 2.11d
*K*_*M*_	43.7 ± 2.36a	51.9 ± 1.63b	55.7 ± 5.70b,c	62.7 ± 6.89b,d
*R* ^2^	0.9979	0.9993	0.9849	0.9920
RPDM	7.54	5.86	8.56	6.48
*v*_max⁡_/*K*_*M*_	1.58	1.27	1.05	0.81

**Table 2:** Statistically significant (*P* < 0.05) values (means ± standard errors) of the parameters in the overall modified Michaelis-Menten models (5), (6), and (7) for the enzymatic reactions of the three amylolytic enzymes in unaged starch and in starches aged for different times (24 h, 48 h, and 100 h). The mean values within rows followed by the same letter are not significantly different (*P* < 0.05) from each other. SEE: Standard error of estimate of the model.

	Termamyl		
Parameters	Model (5)	Model (6)	Model (7)

*v*_ max_	37.2 ± 1.39a	37.8 ± 1.28a	37.8 ± 1.28a
*K*_*M*_	35.7 ± 4.41a	35.7 ± 6.18a	35.6 ± 5.70a
*K*_*t*_	0.003 ± 0.0010	0.004 ± 0.0011	189.93 ± 46.14
*R* ^2^	0.9881	0.9901	0.9914
RPDM	9.18	8.83	8.53
SEE	1.11	1.06	1.00

	San Super		

Parameters	Model (5)	Model (6)	Model (7)

*v*_ max_	33.9 ± 0.94a	34.4 ± 0.94a	35.0 ± 1.06a
*K*_*M*_	35.7 ± 3.18a	35.7 ± 6.42a	35.6 ± 9.46a
*K*_*t*_	0.005 ± 0.0010	0.007 ± 0.0011	101.51 ± 18.15
*R* ^2^	0.9944	0.9922	0.9866
RPDM	6.42	6.74	8.51
SEE	0.67	0.78	1.05

	BAN		

Parameters	Model (5)	Model (6)	Model (7)

*v*_ max_	71.1 ± 3.44a	71.7 ± 1.89a	72.5 ± 1.89a
*K*_*M*_	51.2 ± 7.07a	51.2 ± 3.86a	51.2 ± 6.68a
*K*_*t*_	0.003 ± 0.0007	0.004 ± 0.0008	191.87 ± 37.55
*R* ^2^	0.9938	0.9946	0.9948
RPDM	8.40	8.22	8.21
SEE	1.44	1.33	1.39

**Table 3:** Comparison between the values of *v*_max⁡_ and *K*_*M*_ obtained with the overall models (5), (6), and (7) (Table 2) and those obtained with the classical Michaelis-Menten model (1) for the enzymatic reactions of the three amylolytic enzymes in unaged starch (Table 1). *Significant differences for *P* < 0.05. D.F.: Degrees of Freedom.

	Termamyl		
Parameter	Model (5) versus model (1)	Model (6) versus model (1)	Model (7) versus model (1)

*v*_ max_	*t* value = 0.000	*t* value = −0.588	*t* value = −0.588
	*P* value = 1.000	*P* value = 0.588	*P* value = 0.588
	D.F. = 4	D.F. = 4	D.F. = 4

*K*_*M*_	*t* value = −0.765	*t* value = −0.608	*t* value = −0.619
	*P* value = 0.487	*P* value = 0.576	*P* value = 0.569
	D.F. = 4	D.F. = 4	D.F. = 4

	San Super		

Parameter	Model (5) versus model (1)	Model (6) versus model (1)	Model (7) versus model (1)

*v*_ max_	*t* value = 0.000	*t* value = −0.669	*t* value = −1.377
	*P* value = 1.000	*P* value = 0.540	*P* value = 0.241
	D.F. = 4	D.F. = 4	D.F. = 4

*K*_*M*_	*t* value = −0.432	*t* value = −0.268	*t* value = −0.174
	*P* value = 0.688	*P* value = 0.802	*P* value = 0.870
	D.F. = 4	D.F. = 4	D.F. = 4

	BAN		

Parameter	Model (5) versus model (1)	Model (6) versus model (1)	Model (7) versus model (1)

*v*_ max_	*t* value = −1.044	*t* value = −2.156	*t* value = −2.772
	*P* value = 0.355	*P* value = 0.097	*P* value = 0.050
	D.F. = 4	D.F. = 4	D.F. = 4

*K*_*M*_	*t* value = −1.743	*t* value = −2.871	*t* value = −1.834
	*P* value = 0.156	*P* value = 0.045*	*P* value = 0.141
	D.F. = 4	D.F. = 4	D.F. = 4

**Table 4:** Statistically significant (*P* < 0.05) values (means ± standard errors) of the parameters in the Michaelis-Menten model (1) for the enzymatic reactions of the three amylolytic enzymes in starches mixed with different initial gelatine [*G*] concentrations (0, 25, 50, and 75 g/L). The mean values within rows followed by the same letter are not significantly different (*P* < 0.05) from each other.

	Termamyl			
Parameters	Without gelatine	[*G*] = 25 g/L	[*G*] = 50 g/L	[*G*] = 75 g/L

*v*_ max_	36.1 ± 0.56a	32.8 ± 0.61b	30.0 ± 0.50c	26.7 ± 1.39d
*K*_*M*_	30.6 ± 1.38a	31.5 ± 1.71b	35.7 ± 1.60c	40.4 ± 1.61d
*R* ^2^	0.9986	0.9980	0.9987	0.9908
RPDM	6.77	2.16	1.62	5.26

	San Super			

Parameters	Without gelatine	[*G*] = 25 g/L	[*G*] = 50 g/L	[*G*] = 75 g/L

*v*_ max_	33.9 ± 0.83a	32.8 ± 0.56b	30.6 ± 0.78c	27.2 ± 1.06d
*K*_*M*_	31.6 ± 2.25a	36.3 ± 1.79b	43.2 ± 2.81c	47.3 ± 4.60c,d
*R* ^2^	0.9965	0.9983	0.9972	0.9939
RPDM	9.12	10.14	9.49	6.77

	BAN			

Parameters	Without gelatine	[*G*] = 25 g/L	[*G*] = 50 g/L	[*G*] = 75 g/L

*v*_ max_	80.0 ± 2.28a	75.0 ± 3.89b	69.4 ± 4.06c	59.4 ± 4.00d
*K*_*M*_	52.1 ± 3.64a	65.8 ± 7.72b	83.3 ± 10.29c	98.0 ± 13.17d
*R* ^2^	0.9970	0.9922	0.9922	0.9914
RPDM	2.54	7.08	7.33	9.27

**Table 5:** Statistically significant (*P* < 0.05) values (means ± standard errors) of the parameters in the overall modified Michaelis-Menten models (5), (6), and (7) for the enzymatic reactions of the three amylolytic enzymes in starches mixed with different initial gelatine [*G*] concentrations (0, 25, 50, and 75 g/L). The mean values within rows followed by the same letter are not significantly different (*P* < 0.05) from each other. SEE: Standard error of estimate of the model.

	Termamyl		
Parameters	Model (5)	Model (6)	Model (7)

*v*_max⁡_	37.2 ± 0.61a	37.2 ± 0.67a	37.2 ± 0.72a
*K*_*M*_	32.4 ± 4.31a	32.4 ± 4.69a	32.4 ± 5.22a
*K*_*G*_	0.005 ± 0.0007	0.005 ± 0.0008	154.5 ± 26.82
*R* ^2^	0.9963	0.9955	0.9964
RPDM	4.92	5.17	4.90
SEE	0.59	0.65	0.58

	San Super		

Parameters	Model (5)	Model (6)	Model (7)

*v*_max⁡_	36.1 ± 1.22a	35.6 ± 1.17a	36.7 ± 1.28a
*K*_*M*_	37.5 ± 4.64a	37.5 ± 4.90a	37.5 ± 5.29a
*K*_*G*_	0.004 ± 0.0008	0.005 ± 0.0011	183.7 ± 47.64
*R* ^2^	0.9949	0.9943	0.9934
RPDM	9.51	9.62	9.82
SEE	0.68	0.72	0.77

	BAN		

Parameters	Model (5)	Model (6)	Model (7)

*v*_max⁡_	87.8 ± 4.11a	88.9 ± 4.22a	89.4 ± 4.33a
*K*_*M*_	65.9 ± 11.90a	65.9 ± 12.23a	65.9 ± 13.73a
*K*_*G*_	0.006 ± 0.0017	0.007 ± 0.0021	109.11 ± 36.17
*R* ^2^	0.9913	0.9906	0.9889
RPDM	8.49	8.55	9.00
SEE	1.77	1.84	2.00

**Table 6:** Comparison between the values of *v*_max⁡_ and *K*_*M*_ obtained with the overall models (5), (6), and (7) (Table 5) and those obtained with the classical Michaelis-Menten model (1) for the enzymatic reactions of the three amylolytic enzymes in unaged starch without gelatine (Table 4). *Significant differences for *P* < 0.05. D.F.: Degrees of Freedom.

	Termamyl		
Parameter	Model (5) versus model (1)	Model (6) versus model (1)	Model (7) versus model (1)

*v*_max⁡_	*t* value = −2.874	*t* value = −3.214	*t* value = −3.327
	*P* value = 0.045*	*P* value = 0.032*	*P* value = 0.029*
	D.F. = 4	D.F. = 4	D.F. = 4

*K*_*M*_	*t* value = −1.921	*t* value = −1.873	*t* value = −1.683
	*P* value = 0.127	*P* value = 0.134	*P* value = 0.168
	D.F. = 4	D.F. = 4	D.F. = 4

	San Super		

Parameter	Model (5) versus model (1)	Model (6) versus model (1)	Model (7) versus model (1)

*v*_max⁡_	*t* value = −2.582	*t* value = −2.053	*t* value = −3.179
	*P* value = 0.061	*P* value = 0.109	*P* value = 0.033*
	D.F. = 4	D.F. = 4	D.F. = 4

*K*_*M*_	*t* value = −1.982	*t* value = −1.895	*t* value = −1.778
	*P* value = 0.118	*P* value = 0.131	*P* value = 0.150
	D.F. = 4	D.F. = 4	D.F. = 4

	BAN		

Parameter	Model (5) versus model (1)	Model (6) versus model (1)	Model (7) versus model (1)

*v*_max⁡_	*t* value = −2.301	*t* value = −2.182	*t* value = −2.089
	*P* value = 0.083	*P* value = 0.094	*P* value = 0.105
	D.F. = 4	D.F. = 4	D.F. = 4

*K*_*M*_	*t* value = −0.689	*t* value = −0.638	*t* value = −0.577
	*P* value = 0.529	*P* value = 0.558	*P* value = 0.595
	D.F. = 4	D.F. = 4	D.F. = 4
